# An Explainable CNN and Vision Transformer-Based Approach for Real-Time Food Recognition

**DOI:** 10.3390/nu17020362

**Published:** 2025-01-20

**Authors:** Kintoh Allen Nfor, Tagne Poupi Theodore Armand, Kenesbaeva Periyzat Ismaylovna, Moon-Il Joo, Hee-Cheol Kim

**Affiliations:** 1 Department of Computer Engineering, Inje University, Gimhae 50834, Republic of Korea; nforallen94@yahoo.com (K.A.N.); kenesbaevaperiyzat7@gmail.com (K.P.I.); 2 Institute of Digital Anti-Aging Healthcare, Inje University, Gimhae 50834, Republic of Korea; poupiarmand2@gmail.com (T.P.T.A.); joomi@inje.ac.kr (M.-I.J.)

**Keywords:** food image recognition, convolutional neural networks, vision transformers, explainability methods, personalized nutrition

## Abstract

Background: Food image recognition, a crucial step in computational gastronomy, has diverse applications across nutritional platforms. Convolutional neural networks (CNNs) are widely used for this task due to their ability to capture hierarchical features. However, they struggle with long-range dependencies and global feature extraction, which are vital in distinguishing visually similar foods or images where the context of the whole dish is crucial, thus necessitating transformer architecture. Objectives: This research explores the capabilities of the CNNs and transformers to build a robust classification model that can handle both short- and long-range dependencies with global features to accurately classify food images and enhance food image recognition for better nutritional analysis. Methods: Our approach, which combines CNNs and Vision Transformers (ViTs), begins with the RestNet50 backbone model. This model is responsible for local feature extraction from the input image. The resulting feature map is then passed to the ViT encoder block, which handles further global feature extraction and classification using multi-head attention and fully connected layers with pre-trained weights. Results: Our experiments on five diverse datasets have confirmed a superior performance compared to the current state-of-the-art methods, and our combined dataset leveraging complementary features showed enhanced generalizability and robust performance in addressing global food diversity. We used explainable techniques like grad-CAM and LIME to understand how the models made their decisions, thereby enhancing the user’s trust in the proposed system. This model has been integrated into a mobile application for food recognition and nutrition analysis, offering features like an intelligent diet-tracking system. Conclusion: This research paves the way for practical applications in personalized nutrition and healthcare, showcasing the extensive potential of AI in nutritional sciences across various dietary platforms.

## 1. Introduction

Food recognition’s emergence in computational gastronomy is an important area with potential applications in diet tracking, nutritional assessment, and food study [1]. Correctly identifying and classifying food has various uses, including portion control, nutritional analysis, Meal Frequency Log (MFLog), and other uses [2]. While there have been minor advancements in using traditional machine learning and deep learning in nutrition, some tasks, such as handling visually complex food presentations, improving fine-grained classification, ensuring scalability for real-time applications, and addressing inconsistencies in food datasets, still require enhanced methods that can potentially improve the current state of the art in food recognition systems [3,4,5]. The current advancement in computer vision, especially the application of deep learning, particularly the CNNs, has enabled the creation of models that can recognize food-related items from images [6,7]. At the same time, architectures like ResNet, DenseNet, and Inception have delivered great feats in image recognition [8,9]. However, some challenges are associated with using CNN, such as its ability to detect only local features [10,11]. Thus, they have a problem obtaining a global context, which is required to distinguish between visually similar foods [11].

Vision Transformers (ViTs) have recently emerged as a promising alternative with the attention mechanisms to capture long-range dependencies in images [12]. With this capability, ViTs have shown higher performance in several computer vision tasks such as image classification, object detection, and segmentation [10,11,12]. Hybrid models, on the other hand, have the potential to meet and surpass the limitations of traditional approaches by merging the strengths of CNNs and transformers [13]. Many factors delay the accurate development of food recognition systems, one major factor being the complexity and inconsistency of food images [14,15]. Models find recognizing and classifying food items difficult because they can appear in different forms, presentations, and lighting conditions [16]. To further complicate this, there is also inconsistency in food recognition image sizes and quality across various datasets, which often causes biases and potentially twists the model’s classification performance [17,18]. Efficient knowledge transfer is another significant challenge, even though pre-trained modes can significantly improve accuracy and generalization across diverse food recognition datasets [19,20,21]. However, food recognition tasks require considerable computational resources, memory, and skill to finetune these pre-trained models accurately, and scalability is also of great concern [22,23].

Besides preventing disease, nutrition has a significant role in treating and managing various health issues [6]. According to a report from the American Diabetes Association, 2020, medical nutrition therapy, which is integral to dealing with diseases like diabetes, is based on the principles of nutrition science [7]. Furthermore, the field is growing rapidly due to the combination of genomics and personalized nutrition. Personalized nutrition uses individual genetic information to recommend dietary habits [8]; this could significantly impact disease prevention and health improvement.

The field of nutrition has traditionally relied on observational studies and clinical trials, but the advent of AI has supplanted this. These technologies, including machine learning, deep learning, and data analysis, have the potential to uncover complex relationships in large datasets, identify patterns, and generate actionable information [9,10]. From dietary personalization to preventative models that predict disease, the potential uses of AI in nutrition are numerous and widespread. Integrating AI applications in nutrition facilitates the technological advancements reshaping the landscape of dietary interventions [11]. AI techniques hold immense promise in this data-driven era for revolutionizing how we understand, monitor, and optimize nutritional outcomes.

In nutrition, AI can be defined as applying conceptual algorithms, machine learning, and deep learning techniques to analyze, interpret, and make informed decisions from various datasets related to nutritional data, dietary patterns, and other health factors [11,12,13]. Leveraging machine learning algorithms, random forests can be used to analyze genetics and dietary data to understand how nutrients influence human genetic variations [14,15]. Moreover, collaborative filtering techniques are widely used in personalized nutrition recommendations, while deep learning methods such as convolutional neural networks (CNN) and transfer learning pre-trained models (Resnet, EfficientNet, …) assist in identifying and classifying meals using food images to detect dietary patterns and, further, to assess nutritional content [16,17,18].

In this research, we adopted a hybrid model architecture to address these challenges by combining the strengths of CNNs and ViT. We use efficient knowledge transfer using pre-trained weights and advanced training techniques to enhance scalability and efficiency. Our hybrid model architecture uses ResNet50 [24] as the backbone with transformer encoders, combining CNN feature extraction capabilities with the transformer’s attention mechanisms [25]. We aim to capture both local and global features in diverse food images, thereby improving recognition accuracy and robustness.

Pretrained weights provide an initial foundation for the model, which helps for effective knowledge transfer [1,19,20]. To adapt the model to the nuances of different food categories, we fine-tuned these pre-trained models on five different food recognition datasets [26]. We used mixed precision [27] and distributed training [28] to accelerate the training process and efficiently handle large datasets. Mixed precision training performs computations in half-precision while maintaining accuracy, reducing memory usage, and speeding up the training process [29,30,31]. Distributed training balances the model across multiple GPUs for efficiently handling large datasets [32]. At the same time, gradient accumulation allows us to use larger batch sizes even with limited GPU memory, improving training stability [32,33,34]. We evaluate our model on five diverse food recognition datasets. This research is needed to design diet monitoring systems that may be very useful in the fight against lifestyle diseases such as obesity, diabetes, hypertension, and many others [2,35]. Our work also brings a sense of hope for better diet improvement and, therefore, a more optimistic future for this technology. Besides protecting consumers in the food industry, food recognition is a useful tool for this goal [2,35,36,37].

We target food recognition to push the existing state-of-the-art datasets to be more consistent and improve fine-grained classification capability [38]. We additionally approach state-of-the-art food recognition by identifying the gaps in existing methods and providing a strong, effective, accurate alternative. We seek to establish new state-of-the-art food recognition technologies by fusing CNNs and ViTs, pre-trained weights, and advanced training techniques.

The primary purpose of this study is to develop a robust hybrid model that combines CNNs and Vision Transformers (ViTs) to address the challenges identified in food image recognition. Achieving this goal has led to several key contributions, which can be summarized as follows:

- Developing a hybrid CNNs and transformers-based model for efficient food recognition
- Enhance model accuracy and address the computational resources required for Vision Transformers using advanced training techniques such as mixed precision and distributed training.
- Exploring the capabilities of explainable AI techniques (Grad-CAM and LIME) to ease the understanding and interpretation of the machine learning models’ decision-making process
- Integrating the developed model into a mobile application for real-time food monitoring, tracking, and dietary assessment tasks.

Our work advances computational gastronomy and nutrition analysis [2,35] to the wider community by tackling challenges associated with the variability of food images, dataset inconsistencies, and effective knowledge transfer. The solution provides a solid foundation for rapid and precise food image recognition, which may be used in dietary monitoring, dietary assessment of energy value in real-time, and food-related research [2,35,36,37].

This work is organized into sections, with related works in food recognition in Section 2. Section 3 presents a description of our proposed approach, and the experimental results and discussion are reported in Section 4. Section 5 is the conclusion and future work of the study.

## 2. Related Works

Food recognition has seen advancements with deep learning techniques, particularly CNNs and, more recently, Vision Transformers (ViTs). However, the increasing complexity and diversity of food items require finer-grained recognition [39,40], leading to the birth of many methods in this direction for higher accuracy and efficiency in recognition tasks.

### 2.1. CNN-Based Approaches

CNNs, with their ability to capture and extract hierarchical features from images, form the basis for early prevalent models in food recognition [41]. However, although effective, capturing long-range dependencies and global contextual relations within an image has been challenging for CNNs [42]. Tasks that require distinguishing between images with visual similarities, such as food recognition with complex dishes where ingredient interaction and context are crucial, make things more difficult for CNNs [41,42].

Rahmat et al. [43] applied transfer learning with AlexNet to adapt to pre-trained models that were used for recognizing specific cultural Malaysian food. However, their approach struggles with nuanced distinctions, which are needed for complete categories. It could not capture global contextual relationships even though they were able to reduce training time and use transfer learning. In the same light, Rahmat and Kutty [44] used the ResNet-50 architecture with deep residual learning to improve feature extraction even though they still faced challenges, as their approach still could not capture the intricate relationships between ingredients and the overall compassion of the dish.

Other methods, such as high-precision food classification variants of the Inception module [45], like DeepFood and the lightweight CNN [46], demonstrate the ongoing confidence in CNNs for food recognition. However, these approaches still have the limitations of the previous methods used. A multi-task network, developed by Feng et al. [47], called FoodNet could be a good approach. However, as the added complexity of multi-scale and label dependency learning can hinder performance in real-time or large-scale applications, they also illustrate scalability and efficiency challenges. Lastly, still built on CNN architectures like ResNet50, ResNet101, and SENet, Min, W. et al. [48] used the Progressive Region Enhancement Network (PRENet) with enhancements through progressive training and self-attention mechanisms, which was tested on the large-scale Food2K dataset. Prenet could handle fine-grained food recognition and showed strong performance across multiple benchmarks. Nonetheless, integration into resource-constrained systems may be further hampered by challenges in identifying items of diverse categories that appear visually similar and present high computational demands for training. Despite its few shortcomings, PRENet proves good scalability and generalization across various food-related tasks, making it an important contribution to food recognition research.

### 2.2. ViT-Based Approaches

The introduction of ViTs has significantly changed food recognition methods and offers a new approach to modeling relationships within an image. Unlike CNNs, which capture just local features, ViTs capture global dependencies and understand the broader context of an image. This makes them useful, particularly in distinguishing complex dishes and fine-grained details tasks.

Bianco et al. [49] introduced the use of ViTs for food recognition, and these models armed us with the capacity to overcome some of the challenges inherent in CNNs. However, while ViTs are powerful in modeling long-range interactions, their reliance on large amounts of data and computational resources can be a significant drawback, particularly in resource-constrained environments. Similarly, the approach taken in Min et al.’s ISIA Food-500 dataset study [50] used a stacked global-local attention network inspired by transformer architectures. While this method proves proficient at providing a balance between global and local features and detecting foods from different viewpoints, there might be some drawbacks inherent in characterizing fine-grained differences between visually similar food items. The study by Peng et al. [51] extends ViT-based methods, and was based on self-supervised learning (SSL). This study compares various SSL models, including DINO (DIstillation with NO labels ViT-based), against traditional and non-contrastive approaches. Their results highlight ViTs’ ability to learn visual representations from unlabeled food images. Nevertheless, the models’ applications are optimized for batch sizes and computational ability and requires longer training time. Presumably, ViTs have unique benefits, but these outcomes pose some questions regarding resource utilization concerns and generalization capabilities.

ViT-based models are a promising direction for food recognition because they can consider the entire image and the relations between the different segments. However, due to a significant requirement for computation power and shortcomings in capturing subtle differences, which may require complex methods to deal with, hybrid approaches are needed to integrate the strengths of both CNNs and transformers.

### 2.3. Hybrid Approaches

Hybrid models, which integrate CNNs with transformers or other advanced mechanisms, are promising advancements in food recognition that use both approaches’ strengths.

The Multi-Task Guided Multi-View Attention Network (MVANet) developed by Liang et al. [52] takes a multi-task approach alongside multi-view attention for better identification of Chinese food dishes, their ingredients, and associated recipes. MVANet uses multi-view attention together with CNNs to gain a better view of the various features across tasks, as it delivers higher feature complexity compared to the traditional CNN. Nonetheless, this enhances the complexity of the model, making it less scalable or computationally tractable, especially in real-time domains or with large data sets. The second type of hybrid approach is the RES-VMAMBA, a model derived from residual learning and state space models. The second model is the hybrid model that was proposed by Chen et al. [53] and enhances fine, medium, and coarse-grain classification, having both local and global feature extraction techniques. Nevertheless, due to its intricateness, RES-VMAMBA may have the drawback of decreased extensibility and inability to encode transformers at their full capacity for modeling long input-output dependencies. Moreover, the joint learning model for the multi-task setting proposed by Liu et al. [54] enhances CNN-based learning with attention mechanisms to identify the food items and their ingredients simultaneously. On the one hand, the use of CNNs allows this approach to capture the food/ingredients pairing adequately; however, it may not fully leverage the global score and struggles with capturing dependencies, especially for the numerous ingredient dishes. A common approach to enhancing the utilization and modeling of the food recognition dataset is to adapt CNN and ViT models, and their limitations are evident in the Hybrid models. Nonetheless, it appears that the approaches pose several challenges concerning complexity, scalability, and completely capturing complex food content, meaning that more improvements are achievable in the future.

From the above-related studies, it can be observed that regardless of the employed approaches (CNN, ViT, or Hybrid), some limitations can still be identified in food recognition methodologies. We can clearly state that the traditional CNN-based food recognition processes fail to address long-range dependencies. In contrast, though computationally costly, ViTs consider global contexts and entail large-scale datasets. CNN/ViT combinations offer good results, but encoding the data in this way is problematic regarding scalability, complexity, and, most importantly, real-time response performance. These limitations require a solution that balances local and global feature extraction while optimizing computational efficiency, particularly for real-time mobile applications. Our proposed approach addresses these gaps by combining CNN and ViT strengths with optimization techniques for efficient, scalable food recognition.

## 3. Methodology

This section presents the approach to developing the proposed food image recognition model using an integrated CNN and vision-based model. The methodology is structured into several subsections, beginning with the data preparation process (datasets and preprocessing). Then, we discuss the proposed architecture detailing the chosen ViT and the CNN combined to form the proposed hybrid model. The training process is subsequently described, focusing on distributed and mixed precision techniques.

### 3.1. Data Preparation

#### Dataset Selection and Preprocessing

This study merged five datasets to create a dataset with diverse cultural backgrounds and cuisines for robust food image classification.

The Benchmark for Food Image Recognition (ETH Food101): The first benchmark in food image recognition was prepared with the ETH Food101 dataset [55]. It has 101,000 images and 101 food categories of Western and Asian cuisines, making it one of the most common databases in this area. One criterium of this dataset is its deliberately noisy labels in the training set. In real-world situations, data are frequently not well labeled; hence, this noise can mimic such situations, triggering our models to be robust. Additionally, all images are resized to 512 × 512 pixels. Given that Food101 has been widely used in research, it is considered a standard benchmark that paved the way for more specialized datasets to follow.

Cross-Cultural Food Recognition (VireoFood-172): Even though Food101 captured a range of common food items, the VireoFood-172 dataset [56] extends the collection to include other culturally relevant dishes, such as those in Asian cuisine. This dataset, containing 172 categories, tries to bridge the gap in current Western-centric datasets such as Food101, which lacks a range of diverse food items. These foods are generally eaten in Asian countries, so the VireoFood-172 dataset is appealing for cross-cultural food understanding. It attempts to push the limits of model generalization by increasing diversity in the food classes (including classes that are visually similar yet represent entirely different dishes). Therefore, VireoFood-172 becomes necessary to develop models suitable for a global setting.

Focusing on Chinese Cuisine (CNFOOD 241): The CNFOOD 241 [57] is a unique and specialized Chinese food recognition dataset with over 241 diverse food classes from Chinese cuisine. One of the special features of Chinese food is that it looks visually complex; a single dish can hold many ingredients, each adding to the image. However, with that increased complexity comes difficulty in food recognition for models since they must distinguish foods that can sometimes look similar or look like any other foods. CNFOOD 241 solves this problem by making available a concentrated dataset that researchers can use to tune their models to identify Chinese dishes better. This uniqueness is useful to advance further food recognition targeting Chinese cuisine.

A Regional Perspective with Bounding Boxes (UEC-256): This dataset is unique because it offers a regional-specific viewpoint, as the images come from Japanese food sources. With more than 31,000 images and 256 categories, what is distinctive about this dataset is that it not only provides a list of food items but also the bounding box annotations for each food item within an image. This feature makes UE-256 [58] a classification and localization task that can be performed using the same data. The bounding box annotations available for this dataset are also key in modeling and detecting food items using the images. This is extremely useful when different food items are in a single image, as is the case with bento boxes that are common in Japanese cuisine.

Scaling Up Diversity (Food2k): The last food dataset, Food2k [48], extends the previous ideas to a larger, more varied set of food categories. Some details, like the exact architecture of what is covered under Food2k, can be somewhat varied, but in general, the emphasis is on covering a wider variety of food items and likely includes items more sparsely represented in other datasets. Food2k can be especially beneficial in examining the generalization capacity of models over an extensive diversity of common and rare dishes. Due to the diversity, models trained on Food2k are much better prepared to work in practice, where there can be numerous different kinds of food.

**Figure 1:**
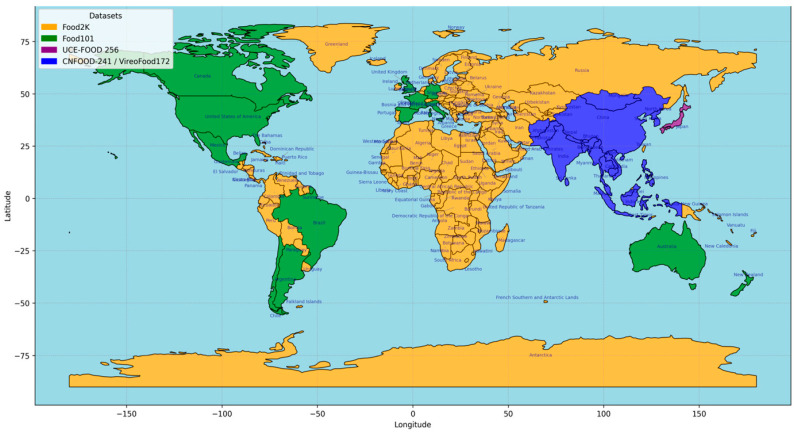
Global representation of food datasets.

**Figure 2:**
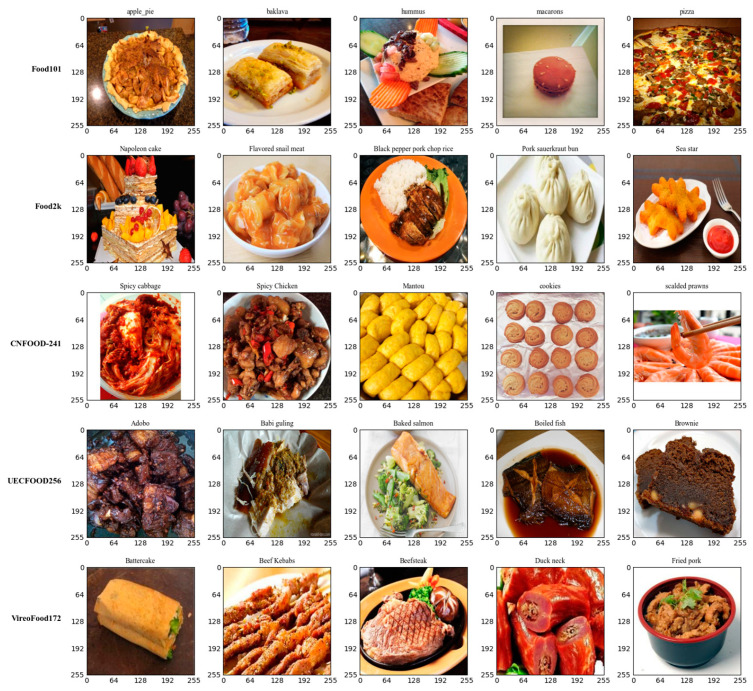
Samples from all the various datasets used.

The above-presented datasets were merged into a single dataset to obtain a larger dataset used in this study, as shown in Table 1. This dataset provides a more comprehensive tool with solid cross-cultural grounds that fairly represents and generalizes the trained models across diverse populations, as indicated in Figure 1. From the initial datasets, we denote $X=\left\{x_{1},\;x_{2},\;\ldots ,\;x_{5}\right\}$ the obtained set of images after merging and $Y=\left\{y_{1},\;y_{2}\ldots ,\;y_{N}\right\}$ the corresponding categories. $x_{1},\;x_{2},\;\ldots ,\;x_{5}$ contains diverse images grouped per category. Each dataset $x_{i},\;i\in \left[1–5\right]$ contains different categories of food images $y_{i},\;i\in \left[1–N\right]$ that are repetitive across the datasets. The merging process consists of creating a single set $Z=\left\{z_{1},\;z_{2},\;\ldots ,\;z_{N}\right\}$ where each image set belongs to a unique category of image food and may result in a fusion of all images of the same category uniquely representing a class. The label set $Y=\left\{y_{1},\;y_{2}\ldots ,\;y_{N}\right\}$ contains N food categories with no duplicates. For the merging process, necessary operations were considered to maintain the dataset’s integrity and avoid bias or false information. All duplicate food categories were carefully analyzed before merging. A manual normalization was conducted on the food categories to obtain the final labels set. Some of the considerations are illustrated in Table 2 below:

Initially, 261 categories had more than one representation in the combined dataset. These categories were finally merged into 142 distinct classes. The obtained dataset resulted in 2651 distinct food categories. Figure 2 shows an overview of the image data contained in the dataset.

Some preprocessing operations were carried out on the Z dataset. First, data were cleaned through duplicate removal, deletion of corrupt files (unreadable images), and filtering of irrelevant images (mislabeled, poor quality, or not food images). All the dataset images were resized to a standard size of 224 × 224 to match the input size requirements and ensure consistency in the input dimension for the model training. Furthermore, data normalization was conducted by pixel scaling, setting all pixel values to [−1, 1] obtained by dividing all pixel values by 255. Additionally, data augmentation operations were implemented to improve model generalization and account for the diversity in the training set using random crop, horizontal flip, and color jittering.

### 3.2. Model Architecture

This research employs a hybrid model made of CNN and vision transformers.

#### 3.2.1. Vision Transformer (ViT)

**Figure 3:**
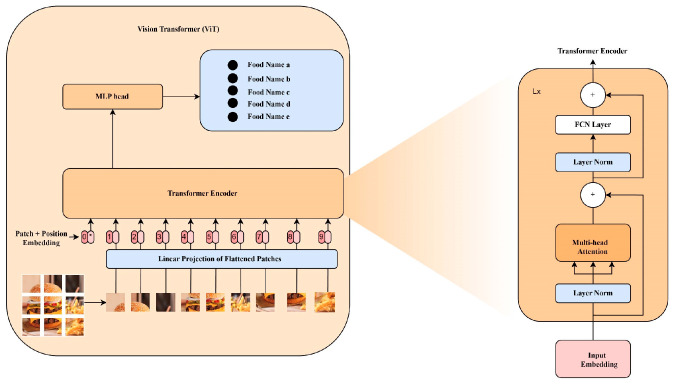
Vision Transformer (ViT) Architecture.

Vision transformers (ViTs) are a novel neural network derived from the transformer model adapted for image processing tasks. Though transformers were developed for natural language processing (NLP), the ViTs leverage a self-attention mechanism to analyze images and predict their class labels [59,60]. This is achieved by representing an input image as a series of image patches like the series of word embeddings used when applying transformers to text. Unlike the basic transformer, ViT uses only an encoder with a multi-layer perceptron for image classification. Figure 3 gives an overview that illustrates ViT, a key component of the hybrid model proposed in this study. This model includes patch embedding, a transformer encoder (made up of MHSA, residual connections, and FNN), and a classification head.

##### Patch Embedding

While CNNs apply filters across the entire image to extract important features, like edges, textures, or shapes, ViT conducts “patchifying”, splitting the input image into smaller fixed-size partitions with no overlap. In the model, each patch is treated as a token, is flattened, and then projected to the output dimension, resulting in a sequence of patch embeddings. Positional information is added to each patch embedding to preserve encoded spatial data. For any input image $x_{i}$ of size, H×W, $\frac{H}{P}\times \frac{W}{P}$ patches of size P are generated and flattened into a 1D vector and projected into higher dimensional space through linear projection. The corresponding output embedding for the $p^{th}$ patch after the linear transformation ($Z_{p})$ has a dimensionality D and is expressed in Equation (1):

(1) $$Z_{p}=Flatten\left(x_{i}\left[p\right]\right).W_{p}+b_{p},$$

$W_{p}$ and $b_{p}$ denote the weight matrix and the bias vector for the $p^{th}$ patch.

The resulting patch embeddings are concatenated to form a matrix $Z\in R^{P\times D}$.

##### Transformer Encoder

The transformer encoder is the main vision transformer’s block that sequentially processes images (herein food images), providing the global contextual understanding necessary for accurate classification. Due to the multitude of food styles, presentations, and backgrounds, coupled with feature scalability, the transformer is an ideal tool. It can effectively capture global dependencies via its self-attention mechanism and process image patches with spatial biases inherent in CNNS. Each transformer encoder block consists of the following:

- Multi-Head Self-Attention (MHSA)

**Figure 4:**
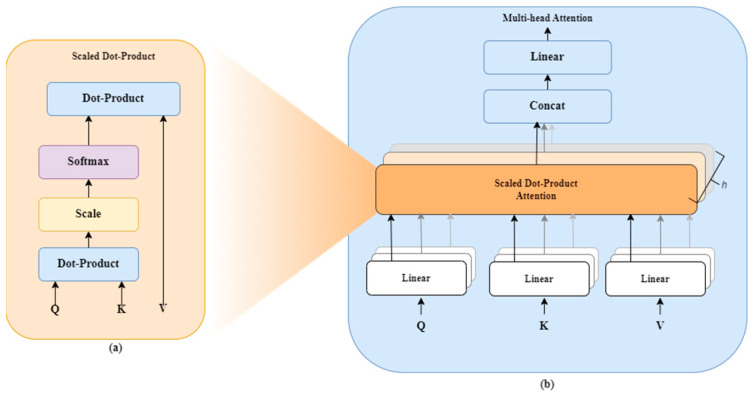
(**a**) Scaled dot-product attention and (**b**) multi-head attention, which consist of serval attention layers running in parallel.

MHSA is the main part of the transformer encoder. The attention mechanism creates interaction between a patch embedding and all other patches. Each patch generates Query (Q), Key (K), and Value (V) vectors used in the self-attention mechanism, allowing the model to weigh the importance of each patch compared to others, as shown in Figure 4. The self-attention mechanism helps ViT capture the image’s global dependencies and distant relationships, thereby overcoming CNN challenges. The attention weights are computed using a scaled dot product expressed in Equation (2):

(2) $$\mathrm{Attention}\left(Q,K,V\right)=\mathrm{Softmax}\left(\begin{array}{c} QK^{T}\\ \bar{\sqrt{d_{k}}} \end{array}\right)V$$

With $d_{k}$ denoting the dimension of the key vectors.

This self-attention mechanism provides a global view that allows the model to handle complex spatial interactions, such as those found in multi-object scenes or the fine patterns visible in food images.

Since there are different representation subspaces at every position, the multi-head attention layer aggregates the various attention heads by concatenation operations, as indicated in Equation (3).

(3) $$\mathrm{MultiHead}\left(Q,K,V\right)=\mathrm{Concat}\left({\mathrm{Head}}_{1},\ldots ,{\mathrm{Head}}_{h}\right)W_{O}$$

where h is the number of attention heads and $W_{O}$ is the output weight matrix.

- Residual connections: This ensures stability during training and gradient flow. For a patch embedding *X*, the output is obtained using the following Equation (4):

(4) $$X_{out}=X+MHSA\left(X\right)$$

- Feed-forward network (FNN): This is a two-layer Multi-Layer Perceptron (MLP) that uses the activation function to transform the output obtained from the attention mechanism. It is generalized and preceded by a layer normalization that ensures training stability. The FFN is computed as shown in Equation (5) below.

(5) $$FFF\left(X\right)=\sigma \left(XW_{1}+b_{1}\right)W_{2}+b_{2}$$

W and b are the trainable parameters.

##### Classification Head

After learning the image representations from the transformer encoder, the classification head is responsible for converting them into class predictions. In our case, this module is responsible for high-accuracy food recognition per category. Generally, the classification head contains a class token CLS representing the last transformer block and is used as a global feature vector. This token is passed through a fully connected layer to map the high-dimensional representation to the number of target classes. The SoftMax activation function generates the logits produced by the linear layer and then converts them into class probability, as in Equation (6).

(6) $$P\left(c_{i}|X\right)=\frac{\exp\left(y_{i}\right)}{\sum_{j=1}^{C}\exp\left(y_{j}\right)}$$

where *C* is the number of food categories.

#### 3.2.2. Transfer Learning

Transfer learning is crucial to this study as pre-trained models provide a strong initialization for the ViT, helping speed up convergence and improve generalization. These pre-trained models helped with a strong feature representation to be fine-tuned to our food classification task because they were trained on large datasets such as ImageNet.

##### Pre-Trained Models Used

In this study, the following pre-trained models were used:

- ViT-B_16: A fine-grained feature extraction of the 16 × 16 patch size ViT model, pre-trained on ImageNet, provides a robust initialization necessary for our task [59].
- ViT-B_32: This is another ViT model with a patch size 32 × 32, which provides an alternative between computational efficiency and fine-grained feature representation.
- R50-ViT-B_16: This research adopts the R50-ViT-B_16 architecture and integrates CNN and ViT to enhance image classification performance, specifically through a hybrid model combining ResNet-50 (R50) and Vision Transformer (ViT-B16).

We begin with a ResNet-50 backbone that serves as a feature extractor and has been modified by incorporating Squeeze-and-Excitation (SE) blocks, which recalibrate channel-wise feature responses to emphasize more informative features. It also includes an initial convolutional block followed by several stages of bottleneck residual blocks. After each bottleneck block, SE blocks are inserted to enhance the model’s adaptive focus on important features. This backbone produces high-dimensional feature maps (2048 channels), crucial inputs to the Vision Transformer component.

Following the ResNet-50 feature extraction, our architecture includes a patch embedding layer to bridge the ResNet’s output with the Vision Transformer’s input requirements, which first reduces the channel dimensionality from 2048 to 1024 using a 1 × 1 convolutional layer. Then, a Conv2D layer with a stride equal to the patch size generates a sequence of patches from these feature maps, which are flattened to form a 2D sequence that serves as the tokens for the Vision Transformer.

A ViT-B16 encoder was used to capture an image’s global information. It starts by prepending a learnable class token to the sequence of patch embeddings and includes positional embeddings so that spatial information among patches is not lost. A transformer encoder of twelve layers was used, each with multi-head self-attention that can simultaneously attend to different parts of the image. Each contains a feed-forward network, and the layers are interleaved with residual connections to increase non-linearity and stable training via layer normalization.

Lastly, the class token is passed through an MLP head for classification with global contextual enriched information. This head contains a densely connected linear layer that maps the class token to a specific number of output classes with a softmax activation giving out probabilities over these classes. The combination of these models (hybrid) has been shown to perform exceptionally well for tasks that need specific texture recognition and high-level image understanding because the ResNet-50 is good in localized feature extraction and the ViT complements it with global context.

**Figure 5:**
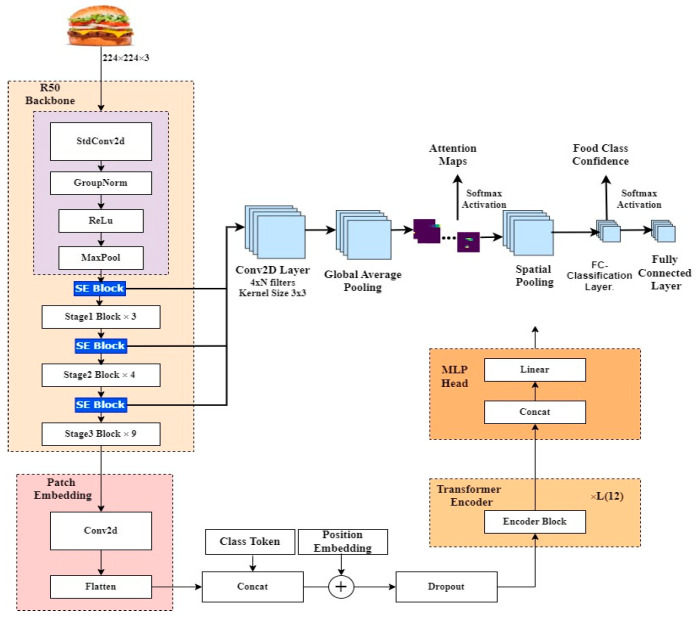
R50 + ViT-B_16 hybrid model.

These pre-trained models are chosen for their balance between computational efficiency and the ability to capture intricate patterns in image data. Figure 5 illustrates the adopted architecture as described above.

##### Model Initialization with Pre-Trained Weights

To ensure the model can immediately start learning meaningful features without learning the basic structures from scratch, the weights for the patch embedding layer *W_p_* and *b_p_* are loaded from the pre-trained model, and positional embeddings *E_pos_*, with initialized values from the pre-trained model, as shown in Equations (7) and (8). This is an important step because it permits our model to retain the special information about the patches essential for accurate image classification tasks.

(7) $$W_{p}=LoadWeights\left(Model=ViT,Layer=PatchEmbeddings\right)$$

(8) $$E_{pos}=LoadWeights\left(ViT,PositionEmbeddings\right)$$

**Figure 6:**
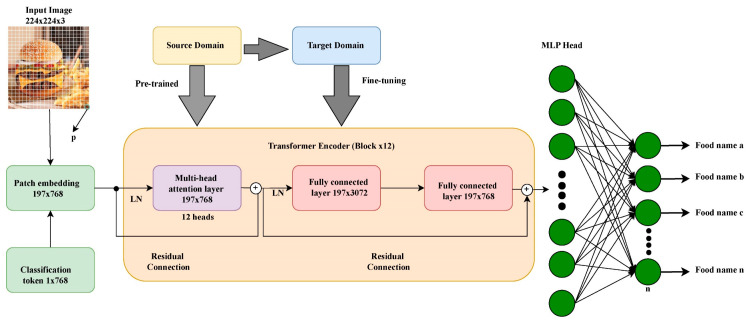
An architectural diagram of the pre-trained models used as a schematic representation of transfer learning.

The pre-trained weights are then used to initialize the transformer blocks, which include the multi-head attention layers and feed-forward networks, as shown in Equations (9) and (10). This entire process, as described, is visually illustrated in Figure 6, where the pre-trained model is loaded to ensure that the model does not learn features from scratch but immediately starts learning meaningful features using the source and target domain, with the MLP head used to classify the different food categories. This helps transfer the knowledge captured by the model during its pre-training on large-scale dataset weights or with weights fine-tuned on a similar classification task. In our case and for models pre-trained on ImageNet, the classification head is adapted to the specific number of classes in each of the five datasets.

(9) $$W_{MHSA}=\left\{LoadWeights\;from\;ViT,\;MultiHeadSelfAttention\right\}$$

(10) $$W_{FFN}=\left\{LoadWeights\;from\;ViT,\;FeedForwardNetwork\right\}$$

##### Fine-Tuning Process

After implementing the pre-trained weights, the model is trained to classify food images. Because extreme updates may disrupt the pre-trained features, the learning rate is lower than the standard training from scratch. A typical choice is shown in Equations (11) and (12).

(11) $$\eta_{fine\text{-}tune}=\frac{1}{10}\eta_{pre\text{-}train}$$

(12) $$\eta_{t}=\eta_{fine\text{-}tune}\cdot Scheduler\left(t\right)$$

To retain their learned representations, the lower layers of the model, as shown in Equation (13), which capture more generic features, may be frozen at the initial stage and gradually throughout to allow the entire model to adapt to the new task; these layers are unfrozen during training. Regularization techniques such as dropout and weight decay, as in Equation (14), are used during fine-tuning to prevent overfitting as the model is fine-tuned on a relatively smaller dataset than its pre-training data.

(13) $$Freeze\left(L_{lower}\right),Unfreeze\;after\;E_{warm\text{-}up}$$

(14) $$L_{reg}=\lambda \overset{P}{\underset{i=1}{\sum }}{\left||W_{i}|\right|}^{2}+Dropout\left(p\right)$$

This fine-tuning process helps the model to adjust its weights to the specific distribution of the datasets while using the rich feature representations learned during pre-training.

### 3.3. Training Procedure

For the training process, we set hyperparameters that will decide how our model can learn. The initial learning rate is set to *η*_0_ = 0.03, and the learning rate scheduler adapts it during training by applying a weight decay term that regularizes the model, as illustrated in Equations (15) and (16).

(15) $$\eta_{t}=\eta_{0}\cdot Scheduler\left(t\right)$$

(16) $$L_{reg}=\lambda \overset{P}{\underset{i=1}{\sum }}{\left||W_{i}|\right|}^{2}$$

Second, the optimizer is stochastic gradient descent (SGD) with a momentum of μ, accumulating gradients over *k* steps before averaging and performing the update. We then clip the gradients not exceeding a maximum norm *γ* to avoid potential instability.

This is properly expressed in Equations (17)–(20).

(17) $$v_{t+1}=\mu v_{t}-\eta \nabla_{W}L$$

(18) $$W_{t+1}=W_{t}+v_{t+1}$$

(19) $$\Delta W=\frac{1}{k}\overset{k}{\underset{i=1}{\sum }}\nabla_{w}L\left(x_{i},y_{i}\right)$$

(20) $$if\;∥\nabla WL∥>\gamma ,\;\nabla WL=\frac{\gamma }{∥\nabla_{W}L∥}\nabla_{W}L$$

#### 3.3.1. Distributed Training

Distributed training is applied to use multiple GPUs. The Distributed Data-Parallel (DDP) framework synchronizes the model parameters across GPUs, with the training loss computed as in Equation (21):

(21) $$L_{total}=\frac{1}{n}\overset{n}{\underset{i=1}{\sum }}L\left(x_{i},y_{i}\right)$$

where *n* is the number of GPUs, and the gradients are averaged across all GPUs.

#### 3.3.2. Mixed Precision Training

Mixed precision training is employed to enhance computational efficiency. The model is trained using a combination of 16-bit and 32-bit floating-point precision. The loss, as shown in Equation (22), is scaled by a factor s to maintain numerical stability, and the gradients are computed with scaled precision and then unscaled before updating the weights.

(22) $$L_{scaled}=s\;\cdot L$$

#### 3.3.3. Training Loop

Our training loop includes a batch size of 32 and an initial learning rate of 0.03 with SGD optimizer at a momentum of 0.9 and no weight decay. This is followed by a warmup strategy of 500 steps, transitioning into a cosine decay of over 100,000 total steps. The gradient accumulation was set to 1 step, and gradients were clipped to a maximum standard of 1.0. Mixed precision training with 16-bit floating-point (FP16) is supported via NVIDIA Apex at optimization level O2. For reproducibility, the training was initialized with a random seed of 42, and we saved the model checkpoints in the specified output directory.

## 4. Results and Discussion

This section will discuss how we set up our proposed approach’s training and testing environments and results and compared its performance with other techniques.

### 4.1. Experimental Setup

#### Environment

For the experiment’s environment, PyTorch in Spyder IDE was used, with APEX for mixed-precision training, allowing the model to use both 16-bit and 32-bit floating-point numbers on a 13th-gen Intel(R) Core (TM) i5-13400 2.50 GHz and 64 GB system RAM with a GeForce RTX 4080 GPUs of 16 GB RAM manufactured by Intel Corporation (Santa Clara, CA, USA). For distributed training, a system equipped with four NVIDIA RTX A5000 GPUs manufactured by NVIDIA Corporation (Santa Clara, CA, USA), each with a computing capability of 8.6, supported by DDP, suggests that the environment can scale across multiple GPUs, possibly spread over multiple nodes.

### 4.2. Evaluation Metrics

Several metrics were used to assess the performance of our model:

- Accuracy: This metric measures the model’s effectiveness by evaluating how many overall predictions were correct. The accuracy is computed as:

(23) $$\mathrm{Accuracy}\;=\left(\frac{TP+TN}{TP+FN+FP+TN}\right)\times 100$$

- Recall: This measures the ability of a model to identify all relevant instances of a specific food class correctly, calculated as:

(24) $$\mathrm{Recall}\;=\frac{TP}{TP+FN}$$

- Precision: That gives the proportion of images classified to a specific class that truly belongs to that class, provided by:

(25) $$\mathrm{Precision}\;=\frac{TP}{TP+FP}$$

- F1 Score: This measures the test’s accuracy, defined as the harmonic mean of precision and recall, computed as:

(26) $$F1\;\mathrm{Score}\;=\left(\frac{2\times Recall\times Precision}{Recall+Precision}\right)\times 100$$

- Top-k Accuracy: A metric that represents how often the correct class is in one of the top-k predicted classes with the highest predictions:

(27) $$\mathrm{Top}\text{-}k\;\mathrm{Accuracy}\;=\frac{1}{N}\overset{N}{\underset{i=1}{\sum }}1\left(y^{\left(i\right)}\in \;P^{\left(i\right)}\right)$$

where *N* is the total number of samples in the dataset, $y^{\left(i\right)}$ is the true class label for the *i*-th sample and $P^{\left(i\right)}$ represents the set of top k predicted classes for the *i*-th sample.

### 4.3. Model Training, Testing, and Evaluation

To ensure a consistent evaluation during the training and testing phases of the pre-trained ViT models used in this study, a standardized set of initial parameters was used, with input image size set to 224 × 224 pixels, a standard ViT requirement. We performed a periodical assessment based on validation accuracy during the training, which was set to stop the training if no improvements were observed using the early stopping criterion based on the validation accuracy. Additionally, the validation dataset was used after a specified number of steps to evaluate the performance of our model. In optimization, a cross-entropy loss function was used, which is appropriate for the multi-classification characteristics of food recognition.

**Figure 7:**
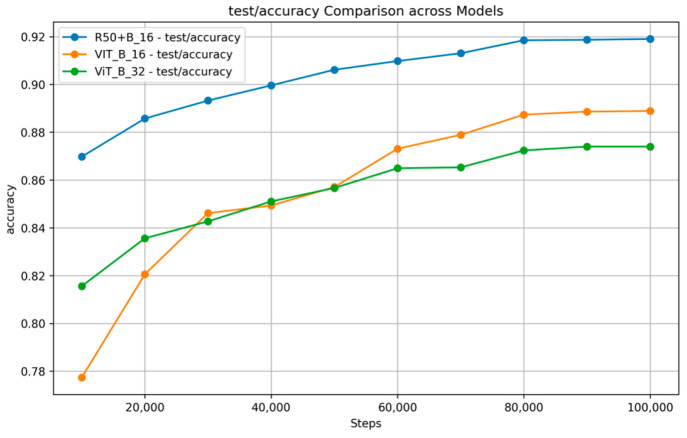
Accuracy Comparison across Models on VireoFood172.

**Figure 8:**
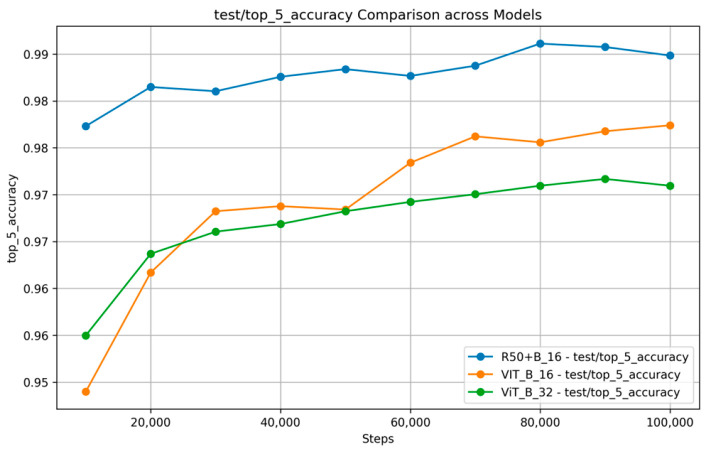
Top 5 accuracy Comparison across models on VireoFood172.

**Figure 9:**
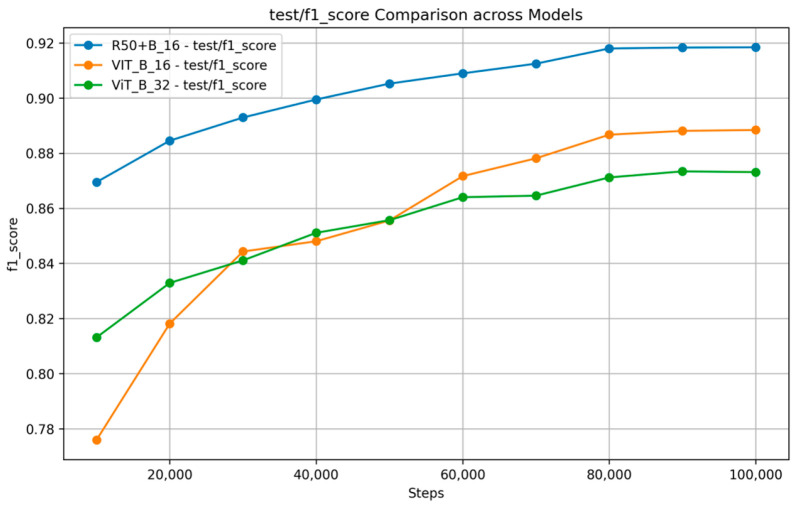
F-1 Score Comparison across Models on VireoFood172.

**Figure 10:**
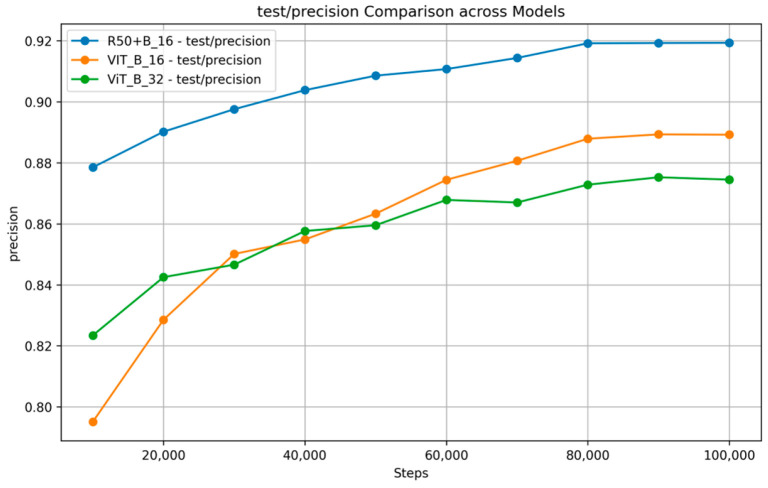
Precision Comparison across models on VireoFood172.

**Figure 11:**
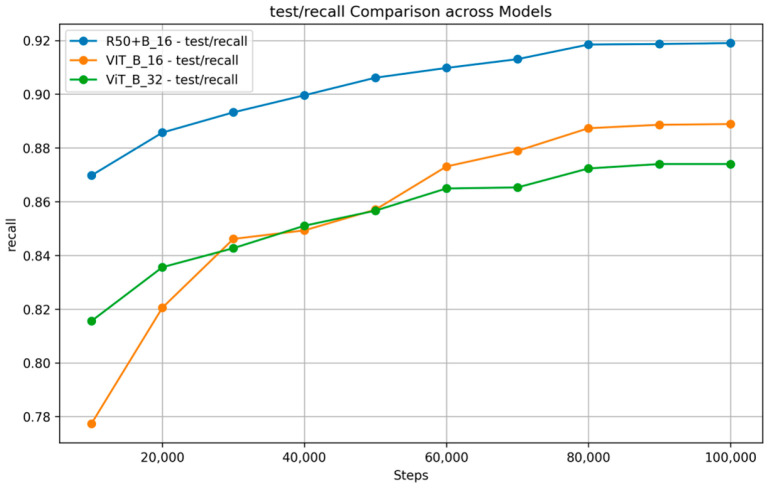
Recall Comparison across models on VireoFood172.

**Figure 12:**
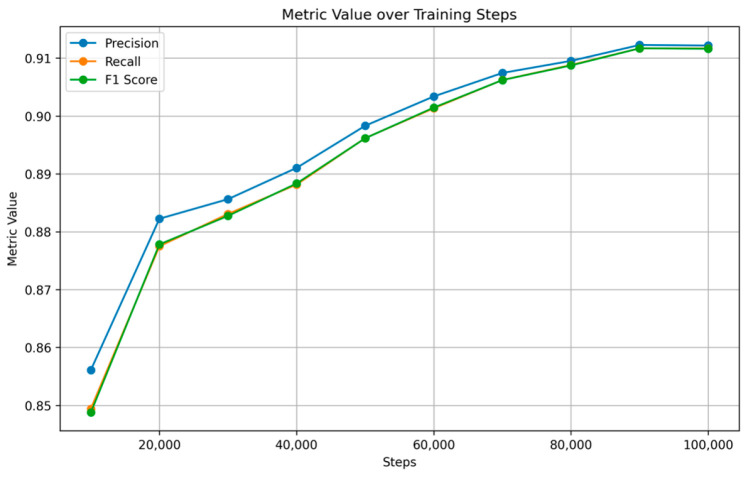
Precision, Recall, and F1 Score on Combined Dataset.

Figure 7 and Figure 8 show the training and validation accuracy and top 5 accuracy curves for each pre-trained model and our proposed model on the VireoFood172 dataset. Figure 7 shows the hybrid model’s steady improvement in training accuracy and superior validation accuracy, showcasing its ability to generalize effectively to unseen data. Furthermore, Figure 8 demonstrates the consistently higher top five accuracy achieved by the hybrid model across steps, reflecting its robustness in identifying the correct class within the top five predictions, even for challenging food categories. We also plotted the F-1 Score, precision, and recall, as shown in Figure 9, Figure 10 and Figure 11, respectively. The combined dataset’s results were evaluated for Precision, Recall, and F1 Score, as shown in Figure 12. These plots provide details into the learning progress, showing how each model adapts to the different datasets used in this study. We divided the dataset into training and validation sets, maintaining an independent validation set to evaluate the model’s performance and generalization, implementing hyperparameter optimizations, and avoiding over-fitting.

It should be noted that the R50 + ViT_B_16 model provides competitive results in terms of accuracy and top five accuracy in all datasets compared to ViT_B_16 and outperforms for these two metrics, whereas ViT_B_32 is always a worse player as shown in Table 3 and Table 4. This demonstrates the hybrid’s performance due to the powerful image representations that stand-alone ViT or CNN architectures could achieve, presumably on the back of the convolutional layers for feature extraction. Our combined dataset uses complementary features from individual datasets to achieve competitive performance and high generalizability, highlighting the importance of dataset fusion in developing robust models capable of addressing global food diversity and variability.

As shown in Table 5, performance comparison across six datasets demonstrates competitive or superior accuracy compared to other works in the field. On the CBFOOD-241 dataset, we achieved an accuracy of 83.4%, which outperforms the results reported in [53]. Similarly, for the UCE-Food256 dataset, our model’s accuracy of 85.0% is substantially higher than the accuracies reported by [44,45]. On the VireoFood172 dataset, we attained an accuracy of 93.7%, which exceeds the performance reported in [47,48,52,54]. This shows the effectiveness of our approach in handling complex food image datasets. In the case of Food101, our model reached 91.3%, showing superior results compared to [44,45,46], while maintaining competitive performance against [48,49]. For the Food2k dataset, our accuracy of 84.1% slightly surpasses the results in [48]. Our model performs robustly across all these diverse datasets, consistently outperforming or matching existing methods.

### 4.4. Explainability

Explainability techniques help understand and interpret machine learning models’ decision-making process, offering insights into which features or regions influence their predictions [61,62]. This work focuses on two popular methods: Grad-CAM and LIME.

### 4.5. Grad-CAM (Gradient-Weighted Class Activation Mapping)

**Figure 13:**
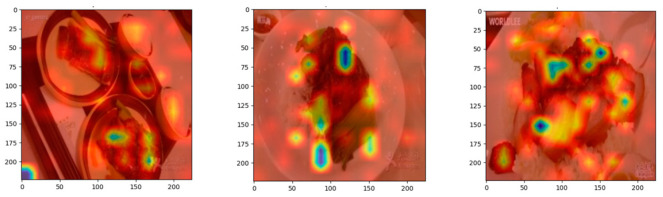
Visualization of Grad-CAM for explainability.

In implementing Grad-CAM for the Vision Transformer (ViT)-based food classification model, hooks were used to capture the feature maps and gradients from a specific layer during forward and backward passes [61]. The gradients are averaged to generate weights, which are combined with the feature maps to create class activation maps (CAMs) highlighting the most important regions influencing the model’s prediction, as shown in Figure 13. For ViT, the class token was excluded to focus only on image patches. The heatmap for a large input image is then resized to the dimensions of the corresponding input image and overlaid on it, such that we get an insight into which areas of a feature in the model played a major role during the decision-making by the model.

### 4.6. LIME (Local Interpretable Model-Agnostic Explanations)

LIME works by generating perturbations of the input image and observing the model’s predictions for each perturbation, thus identifying which parts of the image contribute most to the prediction [63].

**Figure 14:**
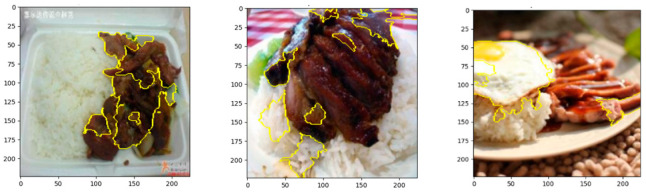
Visualization of LIME for explainability.

LIME generates perturbations of the input image and makes predictions about each perturbation [64]. LIME’s image explainer was used to segment the image using random algorithms (SLIC, Felzenszwalb, or Quickshift), and the perturbations were applied to these segments, as shown in Figure 14. The model predicted class probabilities for each perturb image, which allowed LIME to understand how much each segment contributed to the final prediction. Afterward, we visualize the top contributing regions by overlaying a mask on the original image, highlighting the important areas for the predicted class.

## 5. Model Integration

**Figure 15:**
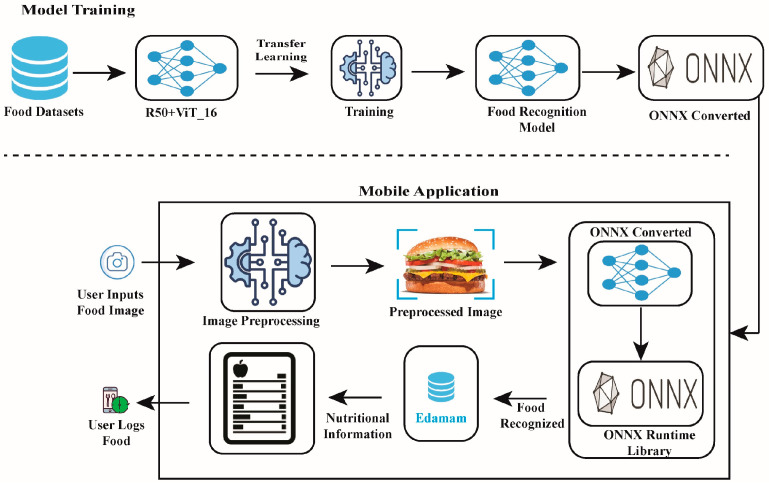
Mobile Application Integration Architecture.

**Figure 16:**
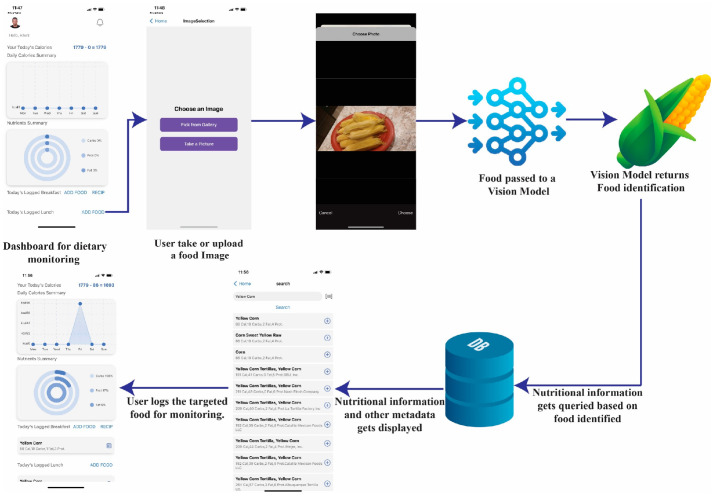
Integration into a mobile dietary monitoring, tracking, and nutritional assessment application.

Subsequently, the trained model was converted into an ONNX (Open Neural Network Exchange), widely used for mobile deployment, to improve its performance on mobile devices. It was then integrated into a mobile application developed in React Native with Expo and Expo Router, as illustrated in Figure 15. The app enables users to take a photo of the food or choose a photo from their camera roll. The image captured or uploaded is passed through the model for recognition, which is initiated directly on the mobile application running the interface and does not require a constant internet connection to function. As shown in Figure 16, our trained model has been successfully integrated into a functional mobile application, demonstrating its ability for real-time dietary assessment.

Once the food is identified, the recognized food name is sent to the Edamam database, which contains information including, but not limited to, calories, macros, etc. The extracted nutritional information is then presented to the user, who can record the recognized food in the user’s diary for monitoring. This helps make dietary information easy to follow and understand by the common user, resulting in easy tracking of nutritional habits for improved nutrition. Thus, the application is a comprehensive tool for food recognition and dietary tracking, enhancing the user experience through real-time feedback and accessibility.

## 6. Discussion and Conclusions

This study presented a hybrid food recognition model that integrates Convolutional Neural Networks (CNNs) and Vision Transformers (ViTs) to address critical challenges in food image classification. The hybrid model uses the strengths of CNNs in capturing localized features, such as edges and textures, and ViTs in modeling global dependencies and contextual relationships across the entire image. This combination allows the model to handle complex and diverse datasets with high variability in food presentation, ensuring more accurate and robust recognition. The evaluation conducted on five diverse datasets, including Food101, VireoFood172, CNFOOD-241, UCE-Food256, and Food2k, as well as a combined dataset, demonstrated the hybrid model’s superior performance compared to state-of-the-art methods as shown in Table 4. The model consistently achieved higher accuracy and top five accuracy rates across all datasets. For instance, on the combined dataset, the hybrid model outperformed individual ViT-based and CNN-based models by effectively addressing the limitations of each approach, such as the inability of CNNs to capture long-range dependencies and the high computational cost of ViTs when used independently. The integration of Grad-CAM and LIME explainability techniques is a notable strength of this study. Grad-CAM generated class activation maps, visually highlighting the regions in the image that influenced the model’s predictions. LIME complemented this by segmenting the image into interpretable parts and assessing their contribution to the model’s classification decisions. These techniques provided valuable insights into the model’s decision-making process, enhancing transparency and user trust. Such explainability is essential for applications in personalized nutrition, where users and practitioners must understand the basis of dietary recommendations or assessments. The hybrid model was further validated through its integration into a mobile dietary monitoring and tracking application. This application directly enables real-time food recognition on mobile devices, where users can capture or upload food images processed by the model to identify food items and retrieve their nutritional information. This functionality has significant implications for personalized health management, allowing users to monitor their dietary habits, track nutritional intake, and make informed dietary choices conveniently. In addition to its technical contributions, this study addresses practical challenges in food recognition, such as variability in food presentations, diverse cultural contexts, and inconsistencies across datasets. The combined dataset, created by merging five benchmark datasets, provided a comprehensive and diverse training ground for the model. This approach ensured that the model could generalize effectively across different cuisines and food categories, addressing a major gap in existing food recognition research. This study establishes a new benchmark in food image recognition by integrating CNNs and ViTs into a hybrid framework. The model’s enhanced performance, explainability, and practical implementation in a mobile application highlight its potential as a transformative tool in personalized nutrition and automated dietary systems. By addressing the limitations of existing methods and proposing a scalable, interpretable, and accurate solution, this study lays a strong foundation for future advancements in the intersection of artificial intelligence and food science. Despite its success, several challenges, such as the combined dataset, despite its diversity, may still have limitations in representing certain food categories or cuisines, which could influence the model’s adaptability to unrepresented regions. Moreover, although the hybrid model exhibits scalability through distributed and mixed-precision training, further optimization is needed to enhance its deployment efficiency on resource-constrained devices. Future research should focus on linking food recognition with predictive modeling for health outcomes, such as obesity and diabetes, and integrating it with health monitoring tools for real-time dietary insights. Expanding dataset diversity and optimizing the model for edge computing will enhance accessibility and performance, paving the way for more versatile dietary assessment tools.

## Figures and Tables

**Table 1:** Details of datasets used.

Dataset Name	No of Classes	Total Samples	Regions	Size (GB)	Ref.
Food2K	2000	1,036,564	Miscellaneous	64.2	[48]
Food101	101	101,000	Western	9.55	[55]
VireoFood172	172	110,241	Asia	1.52	[56]
CNFOOD-241	241	191,811	Chinese	9.94	[57]
UCE-FOOD 256	256	31,395	Japanese	3.97	[58]
Total	2770	1,471,011		89.18	

**Table 2:** Normalization of Food Categories Across Datasets.

SN	Normalized Category	Original Food Names	Datasets	Type of Operation
1	beef curry	(“Beef curry” vs. “beef curry”)	(‘VireoFood172’, ‘FOOD 256’)	Case Sensitivity
2	saozi noodles	(‘Saozi noodles’, ‘saozi noodles’)	(‘FOOD 2K’, ‘CNFOOD-241’)	Punctuation
3	rice	(‘Rice’, ‘Rice’, ‘rice’)	(‘VireoFood172’, ‘CNFOOD-241’, ‘FOOD 256’)	Spacing Issues
4	bibimbap	(‘bibimbap’)	(‘FOOD 256’, ‘FOOD 101’)	Uniform

**Table 3:** Accuracy and Top five accuracy evaluation of our approach on six datasets.

Model	Food2k		Food101		VireoFood172		UCE-Food256		CBFOOD-241		Combined	
	Acc	T-5 Acc	Acc	T-5 Acc	Acc	T-5 Acc	Acc	T-5 Acc	Acc	T-5 Acc	Acc	T-5 Acc
VIT_B_32	68.3	91.1	84.3	96.2	88.0	97.2	81.0	95.2	69.0	91.3	---	
VIT_B_16	77.2	95.3	86.6	97.5	90.0	98.7	77.1	94.3	75.2	94.0	---	
R50 + VIT_B_16	84.1	96.2	91.3	99.0	92.3	98.5	85.0	98.0	83.4	95.2	91.17	98.35

**Table 4:** F1 Score, Recall, and Precision evaluation of our approach on six datasets.

Model	Food2k			Food101			VireoFood172			UCE-Food256			CBFOOD-241			Combined		
	F1 Score	Recall	Prec	F1 Score	Recall	Prec	F1 Score	Recall	Prec	F1 Score	Recall	Prec	F1 Score	Recall	Prec	F1 Score	Recall	Prec
VIT_B_32	63.4	64.1	63.3	86.0	86.0	86.0	89.1	89.1	89.0	94.9	94.0	94.9	76.0	76.0	76.7	-		
VIT_B_16	71.5	71.1	71.0	89.1	89.2	89.2	88.2	88.2	88.2	92.5	92.6	92.7	77.0	75.2	75.8	-		
R50 + VIT_B_16	84.2	84.1	84.0	91.1	91.2	91.3	93.7	93.8	93.8	95.3	95.5	95.3	83.4	83.5	83.4	88.35	91.17	91.22

**Table 5:** Performance comparison across five datasets.

Dataset	Ref.	Technique	Accuracy (%)	Ours (%)
CBFOOD-241	[53]	RES-VMAMBA	82.15	83.4
UCE-Food256	[44]	ResNet-50	49.09	85.0
	[45]	Inception	80.7	
VireoFood172	[47]	FoodNet	89.73	93.7
	[48]	PRENet	90.80	
	[52]	MVANet	91.08	
	[54]	Attention Fusion Network (AFN)	89.54	
Food101	[44]	ResNet-50	39.75	91.3
	[45]	Inception	77.4	
	[46]	CNN	77.3	
	[48]	PRENet	91.13	
	[49]	ViT	92.59	
	[51]	Self-supervised	51.0	
Food2k	[46]	CNN	79.7	84.1
	[48]	PRENet	83.75	
	[49]	ViT	80.76	

## Data Availability

The datasets used in this research are publicly available, and the references are in the dataset description.

## Abbreviations

CNN	Convolutional Neural Network
ViT	Vision Transformer
Grad-CAM	Gradient-weighted Class Activation Mapping
LIME	Local Interpretable Model-agnostic Explanations
FP16	16-bit Floating Point
SGD	Stochastic Gradient Descent
DDP	Distributed Data-Parallel
SE	Squeeze-and-Excitation
ONNX	Open Neural Network Exchange
MFLog	Meal Frequency Log
